# Self-Positivity or Self-Negativity as a Function of the Medial Prefrontal Cortex

**DOI:** 10.3390/brainsci11020264

**Published:** 2021-02-19

**Authors:** Alla Yankouskaya, Jie Sui

**Affiliations:** 1 Department of Psychology, Bournemouth University, Poole BH12 5BB, UK; 2 The School of Psychology, University of Aberdeen, Aberdeen AB24 3FX, UK; jie.sui@abdn.ac.uk

**Keywords:** self-prioritisation, emotion prioritisation, medial prefrontal cortex, fMRI, self-positivity bias

## Abstract

Self and emotions are key motivational factors of a person strivings for health and well-being. Understanding neural mechanisms supporting the relationship between these factors bear far-reaching implications for mental health disorders. Recent work indicates a substantial overlap between self-relevant and emotion information processing and has proposed the medial prefrontal cortex (MPFC) as one shared neural signature. However, the precise cognitive and neural mechanisms represented by the MPFC in investigations of self- and emotion-related processing are largely unknown. Here we examined whether the neural underpinnings of self-related processing in the MPFC link to positive or negative emotions. We collected fMRI data to test the distinct and shared neural circuits of self- and emotion-related processing while participants performed personal (self, friend, or stranger) and emotion (happy, sad, or neutral) associative matching tasks. By exploiting tight control over the factors that determine the effects of self-relevance and emotions (positive: Happy vs. neutral; negative: Sad vs. neutral), our univariate analysis revealed that the ventral part of the MPFC (vmPFC), which has established involvement in self-prioritisation effects, was not recruited in the negative emotion prioritisation effect. In contrast, there were no differences in brain activity between the effects of positive emotion- and self-prioritisation. These results were replicated by both region of interest (ROI)-based analysis in the vmPFC and the seed- to voxel functional connectivity analysis between the MPFC and the rest of the brain. The results suggest that the prioritisation effects for self and positive emotions are tightly linked together, and the MPFC plays a large role in discriminating between positive and negative emotions in relation to self-relevance.

## 1. Introduction

Over the past decade, our knowledge about the effects of self- and emotion-relevance on a wide range of cognitive processes has grown substantially. In particular, self-related disorders are typically associated with the dysfunction of the affective aspects of the self [1,2]. Interest in this topic is growing because it bears far-reaching implications for neuropsychiatric [3], neurological [4], and neurodevelopmental disorders [5].

It has long been recognised that self-referential and emotional processes are closely intertwined [6,7,8,9,10]. A particularly strong body of evidence came from studies demonstrating a synchronised activity between the medial prefrontal cortex (MPFC) as a ‘core’ part of the default mode network (DMN) involved in self-referential processing and the insula, part of the salience network, which has been associated with the processing of both self-relevant and emotion information [11,12]. In light of these findings, the interplay between these two areas may be critical for forming and evaluating personal and contextual meanings of emotional information.

Recent trends in cognitive sciences parallel stimulus encoding mechanisms in terms of self-referential and emotion benefits on the facilitation of cognitive processes [13]. This work suggested a substantial overlap between different processing stages of self-referential and emotion information and proposed the MPFC as one of the neural signatures of the shared mechanisms. Indeed, several neuroimaging studies demonstrated the MPFC to be more active when individuals engage in self-referential processing within an emotional context (e.g., self-evaluation of positive and negative words or judging trait adjectives in terms of their personal relatedness) [14,15,16,17,18,19,20].

However, despite the general agreement that the MPFC plays a key role in processing self-related information in the affective domain, the question about how the MPFC handles the shared mechanisms in investigations of self- and emotion-related processing is poorly understood. For example, some studies reported that the effect of self (vs. other) elicited enhanced activity in the MPFC, but this effect was determined by the degree of self-relatedness of words (high or low) while remaining independent of words of emotional valence (positive or negative) [14,15,17]. These findings, however, have not been replicated in a study using an almost identical experimental task because the results could not dissociate whether the activity in the MPFC was due to the main effect of self vs. other-reference or to an interaction of self-reference and valence [21]. In contrast, a recent study that investigated the neural basis of emotional content in self-referential processing by using a personality attribution task and transcranial magnetic stimulation (rTMS) applied to the MPFC did not find the effect of the rTMS along the self-other dimension but only along the affective dimension [22]. The authors suggested that the MPFC plays a pivotal role in a cortical network that supports affective referential reasoning with a crucial function related to negative attribute processing. The lack of consistency across these studies can be partly attributed to a common confound whereby healthy individuals are motivated to establish the positivity of self by cognitively elaborating and emphasising positive features of the self while dissociating it from any potentially negative features. The tendency to evaluate positive traits as more self-descriptive than negative ones [23], making it challenging to investigate negatively-valenced attributes [24], and confounds self-relevance with emotional valence.

Moreover, it is important to note that the limitations of experimental paradigms in previous studies, probing the effects of valence on the processing of self-relevant information, do not allow for unambiguous conclusions about the precise cognitive mechanisms represented by the MPFC. One emerging point of evidence from recent neuroimaging studies is that the MPFC is involved in the self-positivity bias. In a series of fMRI studies, Beer and colleagues [25] reported that the degree to which participants showed self-positivity bias was associated with activity within the MPFC. Furthermore, the activation pattern within this region and its functional connectivity differed depending on an emotional connotation of self-relevance (positive or negative) [26]. Using a new paradigm in which the valence was varied by whether a statement sentence has a neutral, positive, or negative outcome and self-relevance was varied by changing the subject of a sentence from a person’s name to ‘you’; another team observed a significant effect of self-relevance in the positive, but not the neutral or negative sentences in the MPFC [27]. This self-positivity effect has been linked to downstream processes related to the construction and maintenance of the self-positivity bias [28]. Although these findings are intriguing, it is not easy to address the precise cognitive processes linked to the MPFC.

In sum, it is not clear whether the neural underpinnings of self-related processing reflect positive or negative feelings and emotions. We looked into the issue by using an experimental design with tight control over the factors that determine the effects of self-relevance and emotions to overcome the limitations in previous studies. Our experimental design stems from an associative matching procedure in which a basic stimulus (e.g., simple geometric shapes) is associatively tagged to personally significant information (e.g., ‘self’, ‘friend’, or ‘stranger’) or emotionally valenced information (e.g., ‘happy’, ‘sad’, or ’neutral’). Previous studies using this paradigm demonstrated that immediately after these associations are formed, participants favour the shapes associated with self [29,30,31,32,33,34,35] and emotional information [36,37]. These findings provide evidence that perceptual judgments can be enhanced by tagging external stimuli (i.e., shapes) with an internal self-concept or emotion representations (i.e., referred to by personal labels or emotional cues). Current trends in neuroscience suggested that this empirical operationalisation of self-relevance could be linked to specific functional or neural processes and is used as a model of how we make inferences about ourselves [38,39]. Neuroimaging studies using the associative matching procedure reported replicated effects associated with self-representation (self > other) in the MPFC and the posterior temporal sulcus (pSTS) [40,41]. The effects observed in the MPFC in these studies are in line with previous research suggesting that the key functions of the MPFC are to reflect representations of ourselves (for review, see [24]).

By exploiting the advantages of the associative matching procedure, we aimed to test whether the effects of self-relevance overlap with the effects of positive and negative emotions in the MPFC. Participants performed two separate associative matching tasks, one on personal associations and the other on emotion associations. These tasks followed the same experimental design differing only in the stimuli association (person or emotion) we manipulated. Participants were required to judge whether a displayed shape—item stimulus matched or mismatched learned associations in each task. We hypothesised that if positive attributes of self-related stimuli are the driver of self prioritisation, no significant differences will be found by contrasting the effects of self (over others) and positive emotion (over neutral emotion). We tested this hypothesis in three ways. First, we compared the effects of self and emotions using both the whole brain analysis and region of interest (ROI)-based analysis over the MPFC. Second, we assessed the differences in functional connectivity between the MPFC as a ‘core self’ network [39] and the rest of the brain by contrasting these effects. And third, we controlled the effect of valence by contrasting the effects of self and negative emotion.

## 2. Methods

### 2.1. Participants

A total of 21 young Caucasian adults aged between 21–26 (10 males, age M = 23.6, SD = 2.8, range 20–26) were recruited via a mail list across colleges in Oxford. Participants reported no use of psychotropic medications or past diagnoses for psychiatric, neurological disorders and have normal or corrected-to-normal vision size. As a part of the pre-screening procedure, participants performed the Mood and Anxiety Symptom Questionnaire (MASQ), a 77-item self-report questionnaire that assesses depressive, anxious, and mixed symptomatology [42]. Only participants with low scores on each of the 5 subscales were invited to the scanning session (see Supplementary Materials, Prescreening).

Previous fMRI study [41] using the associative matching task in a sample of 16 participants reported a strong effect size dz = 0.64 (paired-sample *t*-test = 3.21, df = 15, Cohen’s d = 1.65) of the effect of self > other in the ventromedial prefrontal cortex (vmPFC) for the whole-brain analysis and Cohen’s d for Psychophysiological Interaction analyses (PPIs) from 1.58 to 2.02. In the present study, we increased the sample size by 30% to ensure that the current sample has adequate power to detect the differences between conditions, connectivity, and links between connectivity and behaviour.

This experiment was approved by the Central University of Oxford Research Ethics Committee (CUREC). All participants provided written informed consent.

### 2.2. Task and Stimuli

**Figure 1:**
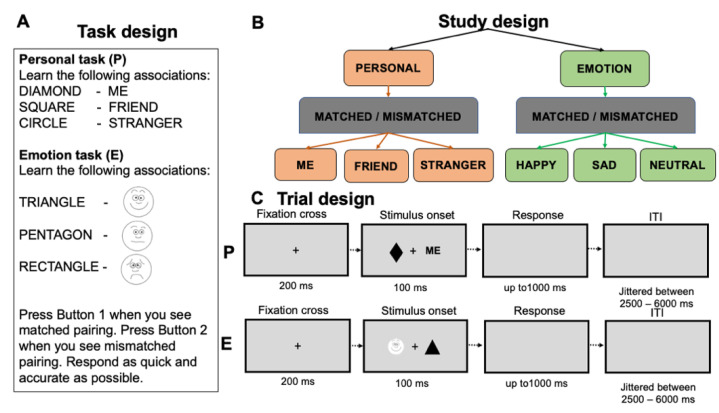
Experimental design (**A**), study design (**B**) and examples of stimuli in the personal and emotional tasks (**C**).

Participants performed two shape-label matching tasks based on personal relevance and emotional valence (Figure 1A). In the personal task, participants were asked to learn three associations between neutral geometrical shapes and personal labels (e.g., diamond—me, square—friend, and circle—stranger). They were required to make a judgment of whether the display contained an associated (matched) or re-paired (mismatched) shape-label combination (e.g., diamond—stranger, me—square) by pressing corresponding buttons on an MRI compatible response box. In the emotion task, participants learned associations between neutral geometrical shapes and schematic faces displaying happy, sad, and neutral emotional expressions (Figure 1A). Emotional schematic faces (i.e., happy, neutral, and sad) were selected from the Wong–Baker Faces [43]. Participants were required to make their responses to matched and mismatched pairings by pressing corresponding buttons similar to the personal task (Figure 1B). Before entering the scanner, each participant performed a short practice with the task (12 trials per task (the number of trials in the training stage was determined by previous studies [33] and a pilot study with 5 participants)). Feedback on accuracy (words ‘Correct!’ or ‘Incorrect!’) and the overall response time was provided after each trial during the practice.

Six geometric shapes (circle, hexagon, square, rectangle, diamond, and triangle) were randomly assigned to three conditions in each task. A stimulus display contained a fixation cross (0.7° × 0.7°) on the centre of the screen with a shape (covering 3.5° × 3.5° of a visual angle) and label (or a schematic face) covering 1.76°/2.52° × 1.76° (3.5° × 3.5°) of a visual angle on either side of the fixation. The distance between the shape and label (or a schematic face) was 10 degrees. Left-right presentations of the shapes and labels/schematic faces were counterbalanced across trials. Each trial started with a fixation cross for 200 ms, followed by the stimulus display for 100 ms and a blank interval that remained for 1000 ms or until the participant responded (Figure 1C). Trials were separated by a jittered interstimulus interval (ranging between 2500–6000 ms). There were 5 runs of 72 trials in each task. The number of trials in the main runs was determined by two previous neuroimaging studies using the associative matching procedure [40,41]. The order of the tasks (PEPEPEPEPE or EPEPEPEPEP) was balanced across participants. Prior to each run, a short instruction reminded participants about their upcoming task. Presentation software (http://www.neurobs.com) was used to present and control the stimuli and collect behavioural measures.

### 2.3. fMRI Data Acquisition and Pre-Processing

fMRI data were acquired on a 3T scanner (Prisma; Siemens) using a 32-channel phased-array head coil. Functional images were acquired with a gradient echo T2*-weighted echo-planar sequence (TR 2040 ms, TE 30 ms, flip angle 80, 64 × 64 matrix, field of view 19.22 mm, voxel size 3 × 3 × 3 mm, parallel imaging GRAPPA, bandwidth = 1628 Hz/Px, PE = 2, and interleaved slice ordering). A total of 36 axial slices (3 mm thick, no gap) were sampled for whole-brain coverage. Imaging data were acquired in 10 separate 180-volume runs of approximately 5 min 10 s each. Whole-head anatomical images were acquired at the beginning of each scanning session using the following parameters: 192 × 192 matrix, voxel size 1 × 1 × 1 mm; TR = 1900 ms, TE = 3.97 ms, and bandwidth = 200 Hz/Px.

*For univariate analysis*, the imaging data were pre-processed using SPM12 (www.fil.ion.ucl.ac.uk/spm). Functional images were realigned, unwarped, slice-timing corrected, and co-registered to the participant’s T1 scan, resliced to a 2 mm^3^ voxel size, and smoothed with a 8-mm FWHM kernel. To account for positive correlations common in the fMRI data and the assumptions made about variance, the serial correlations were estimated with a ReML (restricted maximum likelihood) algorithm using an autoregressive, AR(1), model during parameter estimation. Positive autocorrelation, if ignored, could deflate the estimate of the residual variance and inflate statistical inference [44]. To reduce inter-subject anatomical variability, a high-dimensional diffeomorphic registration (DARTEL) [45] was performed to create an anatomical template at the group level. First, echo-planar images (EPI) were segmented for the brain tissues using the unified segmentation procedure (the ‘new segment’ function) [46]. Flow fields were calculated and subsequently applied to the native grey matter image of each participant. The affine transformation from the group template to the brain template implemented in SPM12 (ICBM152) was computed and applied to each individual grey matter segmentation. The group template was then normalised to the MNI space (Montreal Neurological Institute).

*For functional connectivity analyses*, we used the default pre-processing pipeline implemented in CONN [47] (functional realignment, unwarping, slice-timing correction, structural segmentation and normalisation, functional normalisation, outlier detection, and smoothing). Functional images were co-registered and resliced to a voxel size of 2 mm^3^, normalised to the MNI brain template, and smoothed with an 8 mm isotropic Gaussian kernel. The default pipeline also included head motion artefact detection with the Artifact Detection Toolbox (ART). No images demonstrating head motions of more than 0.9 mm were found in our data. Another set of nuisance regressors were removed from the data using the component-based noise correction technique (CompCor [48]). With CompCor, noise ROIs are defined within the white matter and cerebrospinal fluid (CSF) masks individually for each participant. Then, the signal from the noise ROIs is decomposed with a principal component analysis (PCA), and the resulting component time courses were regressed out from the data. As CompCor addresses potential confounding effects without the risk of artificially introducing anticorrelations into the functional connectivity estimates, global signal regression was not applied to the data. As additional denoising options, we used detrending to remove any linear terms within each functional session and despiking to reduce the influence of potential outlier scans that were not detected by ART. The main effect of the task (i.e., direct BOLD signal changes associated with the presence/absence of a task) was also regressed out by entering the condition main effects as confounds in the pre-processing step. Finally, a standard temporal band-pass filter (0.008 to 0.09 Hz) was applied on the time series to restrict the analysis further to signal fluctuations that characterise the task-based fMRI BOLD frequency band. 

### 2.4. Data Analysis

*Behavioural data.* In each task, we measured accuracy and response times. Here we report data analysis for matched trials only (full data analyses are presented in Supplementary Materials). A one-way repeated measures ANOVA was carried out to examine the effect of stimuli on the response time in each task. We also assessed RT advantages for self-associations compared to stranger-associations in the personal task (self-bias), and RT advantages for happy-and sad-associations (happy- and sad-bias) compared to neutral emotional expression associations in the emotion task.

*fMRI data univariate analysis*. The data were modelled using the general linear model (GLM) implemented in SPM12. First, individual fMRI time series for personal and emotion runs were regressed onto a single fixed-effect general linear model. For the personal runs, the regressors of interest represented matched trials for self, friend, and stranger. For the emotion runs, the regressors of interest were happy, sad, and neutral expressions. Mismatch trials assigned to different response buttons were of no interest for these analyses but included in the general model to account for the model’s BOLD signal variance. The single-subject hemodynamic responses were modelled by convolving delta-stick functions aligned to each condition’s onset with a first-order canonical hemodynamic response function [49]. Stimulus onsets were defined relative to the acquisition of the middle slice. The 6 motion-correction parameters were included in the design matrix to regress out motion-related fluctuations in the BOLD signal. Two temporal derivatives (one controlling for a small shift in the HDR pick, the second controlling for duration dispersion for the pick) [50] were entered as regressors of no interest. Several contrasts were performed at the first-level analysis. Individual contrast maps were entered into a random effect analysis [51] for group statistical interference. The results were assessed with an false discovery rate (FDR)-corrected cluster-wise threshold of *p* < 0.05 based on an uncorrected voxel-wise threshold of *p* < 0.001 and an extended threshold of 30 contiguous voxels. All anatomical coordinates are reported in MNI coordinates. 

*ROI-based analysis in the MPFC*. To test the brain activity in the MPFC associated with biased responses (self vs. others, happy/sad vs. neutral) in each task, we conducted ROI analyses based on the portion of the MPFC where the effect of self has been established by prior studies [41,52] and confirmed in the univariate analysis of the current study.

*fMRI data functional connectivity analysis*. To enhance the interpretability of the univariate analysis and test the hypothesis that the differences between self and emotion biases may be associated with changes in the functional connectivity, we used seed-based connectivity (SBC) analysis. All SBC analyses were performed using CONN v.19b. SBC maps represent the level of functional connectivity between a seed and every voxel or location in the brain. The mean time-series averaged across all voxels within each seed was used as a regression parameter and correlated with all other voxels in the brain in seed-to-voxel connectivity analyses. Before the SBC analyses, BOLD time-series were pre-processed and denoised separately for each run (as described in Section 2.3), then concatenated and normalised to build the BOLD time-series at each voxel and the average BOLD time-series within a seed.

The SBC maps were computed as the Fisher-transformed bivariate correlation coefficients between a seed BOLD time-series and each voxel BOLD time-series. These individual maps were entered into the second-level analysis, where group-level differences in functional connectivity between self and emotion biases were tested. False discovery rate (FDR) corrections were applied to all function connectivity maps [53]. The second-level analysis was performed using paired-sample *t*-tests to compare self and happy/sad biases, with a cluster defining the threshold of *p* < 0.001 uncorrected (cluster size *k* > 30). Results significant at an FDR-corrected cluster level threshold of *p* < 0.05 are reported.

## 3. Results

### 3.1. Behavioural Performance

**Figure 2:**
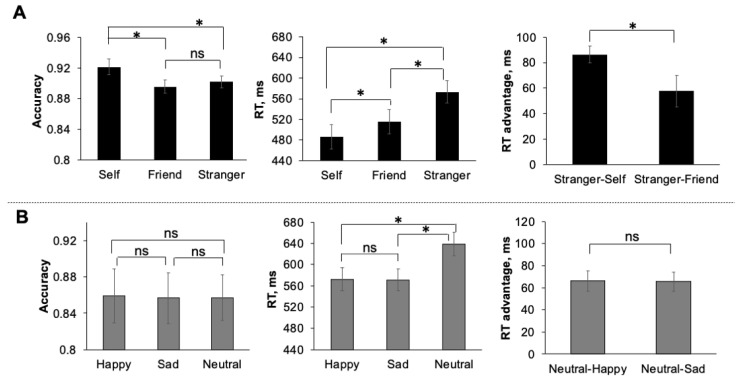
Accuracy, reaction times and RT advantages in personal (raw **A**) and emotional (raw **B**) tasks. Error bars represent ± SEM. * *p* < 0.05.

*Accuracy*. Participants were accurate in both tasks (Figure 2). A one-way repeated measures ANOVA revealed a significant main effect of stimulus in the personal task (F(2,40) = 3.62, *p* = 0.04). Post hoc *t*-tests with adjustment for multiple comparisons using the Holm method indicated that this effect was driven by a significantly higher accuracy for shapes associated with the self, compared to friend (*t* (20) = 2.58, *p* = 0.04, MD = 0.03, 95% CI [0.01, 0.04]). In the emotion task, the main effect of the stimulus was non-significant (F(2,40) = 0.02).

*Reaction time (RT)*. Only correct responses were used for reaction time analyses. Participants were faster (F(2,40) = 27.62, *p* < 0.001) in responding to stimuli associated with self and friend compared to stranger (*t*(20) = −7.32, *p* < 0.001, MD = −88.24, 95% CI [−100.1; −72.71]; *t* (20) = −4.78, *p* < 0.001, MD = −57.66, 95% CI [−82.98; −32.33] respectively). The difference between self and friend was also significant (*t*(20) = −2.54, *p* = 0.02, MD = −30.58, 95% CI [−60.37,−2.84]). In the emotion task, reaction times for happy and sad associations were faster compared to associations with neutral emotional expressions (F(2,40) = 29.70, *p* < 0.001; *t*(20) = −6.83, *p* < 0.001, MD = −69.38, 95% CI [−84.93, −47.35]); *t*(20) = −6.51, *p* < 0.001, MD = −66.14, 95% CI [−82.97; −47.78]). The difference between happy and sad associations were not significant (*t*(20) = −0.32, *p* = 0.75) (Figure 2).

*RT advantages*. To quantify the effects of personal relevance and emotions, we calculated RT advantages (gains) for self and friend compared to stranger ([RTstranger—RTself], [RTstranger—RTfriend]) and for happy and sad emotional expressions compared to neutral ([RTneutral—RThappy], [RTneutral—RTsad]) (Figure 2). The RT advantage for self was significantly greater compared to friend (*t*(20) = 2.70, *p* = 0.01, MD = 39.39.98, 95% CI [8.95, 69.62]). The difference between RT advantage for happy and sad emotional expressions was non-significant (*t*(20) = 0.29).

In parallel to our main hypothesis that self-positivity is an inherent property of self at the neural level, we also explored the associations between prioritisation effects of self, positive, and negative emotions at the behavioural level. Multiple regression analysis was carried out to test whether the magnitude of the prioritisation effects for positive and negative emotions could predict the magnitude of self-prioritisation. Using the enter method, it was found that the prioritisation of positive and negative emotions explained a significant amount of self-prioritisation variance (F(2,20) = 9.04, *p* = 0.002, R^2^ = 0.50). The analysis showed that negative emotion did not predict the effect of self (Beta = 0.18, 95% CI estimated based on 5000 bootstrapping replicates [−0.11, 0.43], t = 1.40, *p* = 0.18). However, RT advantages for positive emotion did significantly predict the magnitude of self-prioritisation (Beta = 0.42, 95% CI estimated based on 5000 bootstrapping replicates [0.17, 0.69], t = 3.43, *p* = 0.003) (for full report, see Supplementary Materials).

### 3.2. Univariate fMRI

**Figure 3:**
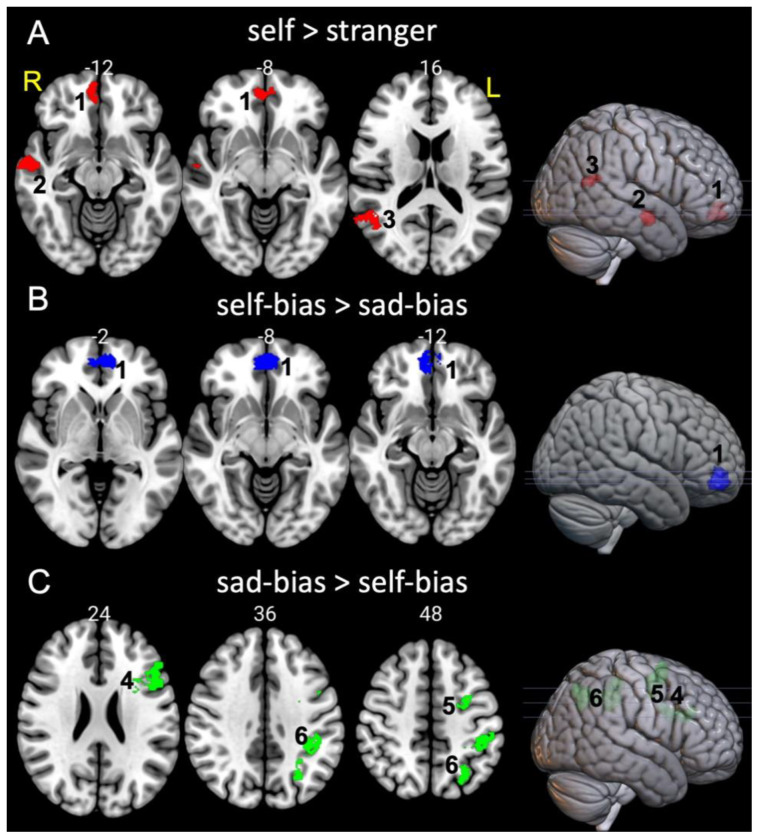
Activation results for the whole-brain univariate analyses for contrasts [self > stranger] (**A**), [self-bias > sad-bias] (**B**), and [sad-bias > self-bias] (**C**). The mask of clusters with significant univariate effects (a cluster corrected FDR-threshold of *p* < 0.05, voxel-threshold *p* < 0.001 uncorrected, extended threshold of 30 contiguous voxels) was created and overlaid on an MNI152 standard template using MRIcroGL (radiological convention).1—vmPFC (ventromedial prefrontal cortex), 2—R-STG (right superior temporal gyrus), 3—R-AG (right angular gyrus), 4—L-IFG-Tri (left inferior frontal gyrus, pars triangularis), 5—L-FMG (left frontal medial gyrus), and 6—L-IPL (left inferior parietal lobe).

First, we tested whether the present study could replicate previous evidence for self-bias effects in the vmPFC and STS. The whole-brain result of the contrast [self > stranger] confirmed the results of previous studies showing that shapes associated with the self elicit greater activity in the vmPFC (k = 213, Z = 4.03, x/y/z = −6/52/−4), the right superior temporal gyrus (R-STG, k = 204, Z = 4.93, x/y/z = 58/−8/−14), and the right angular gyrus (R-AG, k = 138, Z = 3.90, x/y/z = 52/−50/18) compared to shapes associated with stranger (Figure 3A). The contrast [self vs. friend] was not in the focus of the present study. However, we report the results in Supplementary Materials.

We also assessed whether the brain could discriminate between shape-label associations with happy, sad, and neutral emotional expressions. The results of these contrast are displayed in Table 1. No voxels for the reversed contrasts (i.e., [neutral > happy], [neutral > sad]) survived the thresholds.

Second, we performed four contrasts to examine the effects of (i) self-bias [self > stranger] vs. happy-bias [happy > neutral] and (ii) self-bias [self > stranger] vs. sad-bias [sad > neutral]. The results showed that the effects of self-bias did not differ from the effects of happy-bias (no significant clusters survived the threshold) for either [self-bias > happy-bias] and [happy-bias > self-bias]. Contrasting self-bias with sad-bias yields a large cluster in the vmPFC (x/y/z = −6/52/−4, k = 429, Z = 4.19) (Figure 3B). The opposite contrast (sad-bias > self-bias) showed an activation in three clusters (left parietal inferior lobule (L-IPL, x/y/z = −40/−38/40, k = 765, Z = 4.81); the left frontal middle gyrus (L-FMG, x/y/z = −24/−2/44, k = 349, Z = 4.13) with a small portion expanding to the frontal superior gyrus and precentral gyrus; and the left frontal gyrus pars triangularis (L-IFG-Tri, x/y/z = −50/28/28, k = 366, Z = 3.88)) (Figure 3C).

### 3.3. The Effects of Personal and Emotion Associations in the MPFC

To test whether there was increasing (self > friend > stranger) or a decreasing (self < friend < stranger) activity in the vmPFC, a region of interest (ROI) in the vmPFC was created based on the group-level whole-brain analysis for contrast [self > stranger] (Figure 3A). Beta-values were extracted from each participant/condition beta maps, averaged across voxels, and submitted to analyses of variance. To control for the results in the vmPFC, we also selected an ROI in the dorsomedial prefrontal cortex (dmPFC). The involvement of the dmPFC in social evaluative judgements and emotional processing [54,55] are well-established findings. Furthermore, recent studies emphasised some resemblance between the vmPFC and dmPFC in the processing of social and emotional information and questioned a strict functional distinction between these regions during emotional valence processing [56]. Having a functionally similar ROI in the dmPFC as a control provides a unique opportunity here to test whether the effects of personal- and emotion-relevance are specific to the vmPFC. The dmPFC ROI was defined as a 7 mm sphere centred at x/y/z = −6/44/18 [41,54] containing 207 voxels (see details in Supplementary Materials).

**Figure 4:**
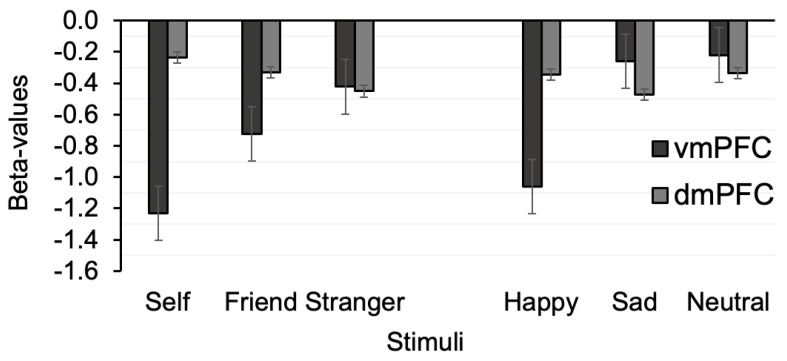
Mean beta estimates in the vmPFC and dorsomedial prefrontal cortex (dmPFC) regions of interest (ROIs). Error bars represent ± SEM.

A one-way ANOVA was carried out separately for each task, and each ROI on means beta-values. In the vmPFC, there was a main effect of Stimulus (F(2,40) = 7.88, *p* = 0.001, η^2^ = 0.28) (Figure 4). Post hoc analyses revealed greater activity for the self compared to stranger (MD = −0.81, Cohen’s d = −0.86, pholm < 0.001) and friend (MD = −0.51, Cohen’s d = −0.54, pholm = 0.04). The difference between friend and stranger was non-significant (MD = −0.30, pholm = 0.15). In contrast, no main effect of Stimulus was found in the dmPFC (F(2,40) = 1.50, *p* = 0.23). In the emotion task, the data indicate a main effect of Stimulus in the vmPFC (F(2,40) = 3.33, *p* = 0.046, η^2^ = 0.14). However, post hoc analysis did not reveal marginally significant differences between happy and neutral (MD = −0.84, t = −2.29, pholm = 0.08) and happy and sad (MD = −0.80, t = −2.18, pholm = 0.08), and in addition, there was no difference between sad and neutral (MD = 0.04, pholm = 0.92). A main effect of conditions in the emotion task was non-significant in the dmPFC (F(2,40) = 1.63, *p* = 0.21).

### 3.4. Seed-To Voxel Functional Connectivity

To further examine self and emotional biases, we tested whether the differences at a cluster level (Figure 3B,C) also reflect differences in functional connectivity between these clusters and the rest of the brain. Although our primary interest focused on the vmPFC, four ROIs were created based on the results of the univariate analysis for contrasts [self-bias > sad-bias] and [sad-bias > self-bias]. All ROIs were created using the utility of Marsbar software (http://marsbar.sourceforge.net/about.html). These ROIs were entered as seeds in a seed-driven functional connectivity analysis to estimate maps showing temporal correlations between the BOLD signal from each seed and that at every brain voxel [47]. Separate analyses were performed for each seed. Response time advantages were entered as continuous regressors within the general linear model at the second level analysis to account for the magnitude of behavioural biases to self and happy/sad associations.

**Figure 5:**
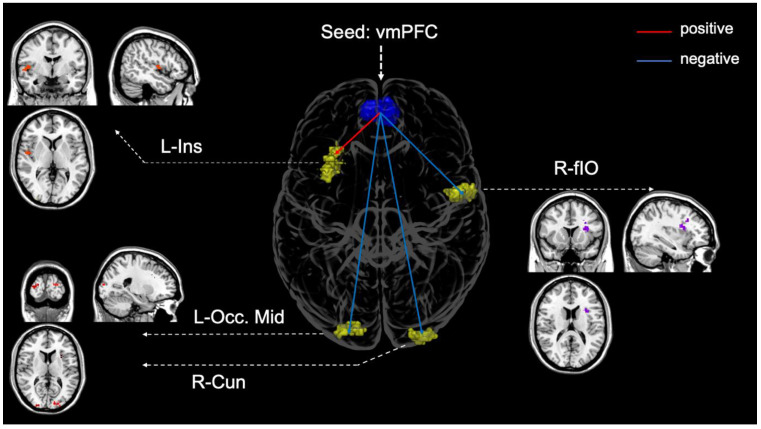
Seed-to-voxel functional connectivity with the vmPFC seed (FDR-corrected at cluster-level with *p* < 0.05, *p* < 0.001 uncorrected height threshold, and 30 voxels extended threshold). Red: Positive connectivity, blue: Negative connectivity.

For the contrast [self-bias > sad-bias], the vmPFC seed was positively correlated to clusters of voxels within the left insula (L-Ins) and negatively to the right frontal inferior opercular (R-fIO), the right cuneus (R-Cun), and the left occipital middle gyrus (L-OCC-Mid) (Figure 5, Table 2).

For the contrast [sad-bias > self-bias], none of these three seeds (Figure 3C) showed connectivity differences above the threshold.

## 4. Discussion

Recently emerging literature have revealed overlapping neural effects of self-relevance and positive emotions, suggesting that these two effects may be inherently related [13,22,24,27,28]. In the present study, we developed this line of inquiry and tested the neural underpinning of self and emotion biases generated by a common experimental procedure [29,30]. Our results support the hypothesis that self-positivity is a key property of self-processing by three findings.

First, the patterns of reaction time performance were qualitatively similar in personal and emotional tasks. In both tasks, items ‘tagged’ with important social information (self, positive, and negative emotions) gained processing priority. These results are in line with previous behavioural studies that reported a consistent advantage for self-related stimuli (compared to stranger-associations) [32,33,34,35] and emotionally valenced stimuli (compared to neutral emotional expression) [36,37,57]. An interesting finding in the present study is that the magnitude of positive but not negative emotion biases could predict the magnitude of the self-prioritisation effect. While preferential processing of positive emotional information in relation to self-relevance is now well-documented in the literature, it is debated as to what extent these two types of preferential processing relate to each other [52,58]. Our finding indicates that, in healthy participants with no negative mood symptoms, prioritising positive emotion is congruent with prioritising self-relevant information. It echoes previous research suggesting that the influence of negative emotion is different from that of positive emotion on the degree of the self-reference effect, specifically, that negative emotional processing, but not positive, weakened the degree of self-reference effects [37,59]. Although intriguing, this finding should be taken with caution. One previous study using a similar experimental paradigm reported no correlation between self and emotion biases [36]. It should be noted that our experimental design differed from the previous study in three ways that could magnify the self-positivity bias. Specifically, (i) we used validated representations of facial expressions instead of schematic faces; (ii) we tested responses to associations with positive, negative, and neutral emotions (in contrast to the previous study [36] where participants were asked to form associations with very happy, happy and neutral emotions); and (iii) we controlled for mood and anxiety/depression symptoms in our sample. The discrepancies between our and previous results highlight an important direction for future research in testing the extent to which the magnitude of self-positivity bias can be enhanced.

Second, our univariate analysis revealed no differences in brain activation between the effects of self and positive emotion prioritisation. However, separately, each effect (self > stranger, happy > neutral) triggered responses in areas previously reported in the literature. In particular, we replicated the effect of self in the MPFC (vmPFC) and the STS/Angular gyrus reported in studies using the associative matching procedure [40,41] and other experimental paradigms to generate a robust self-prioritisation effect [60]. The prioritisation effect for happy emotion in our study elicited activation in the area implicated in the processing of positive emotions and, particularly, smiling faces [61,62]. Thus, the absence of the differences between self and positive emotion is unlikely to result from the lack of these effects per se. One possible explanation may reflect an overlapping effect of self and positive emotion in the MPFC and, particularly, in its the ventral part—the well-known region for its contribution in self-referential processing, emotion regulation, and social cognition [13,39,63]. 

The results of our ROI analysis support this explanation by demonstrating larger effect sizes for self and happy emotion in the vmPFC. Moreover, the analysis also indicates that the overlapping effect may be specific to the vmPFC. Differential involvements of the MPFC in various self-referential and emotional tasks are some of the most frequently observed in functional imaging studies [56,63]. A new finding here is that both self and positive emotion associations yielded equivalent biased responses compared to friend/stranger and neural/sad emotions in the vmPFC, which is part of the default mode network. In contrast, a neighbouring region, the dmPFC, whose contribution to social cognition is also well documented [28,54,56], did not show substantial changes in activity across conditions in personal and emotional tasks. If this finding is confirmed in following up studies, it may indicate that the ventral MPFC plays a role in integrating self-relevant and positive emotional processes by incorporating emotional biasing signals into self-referential processes through the interactions between the DMN and salience network.

Our results indicate that the ventral part of the MPFC, which has established involvement in self-prioritisation effects, was not recruited in the negative emotion prioritisation effect. This finding is in line with previous neuroimaging studies in healthy subjects [28]. It is worth mentioning that neuroimaging work in individuals with mood disorders (which are typically characterised by elevated self-negative ruminations) indicated the opposite involvement of the MPFC [64,65]. For example, in a study where participants were asked to remember parts of their lives related to positive (happiness) and negative (anger) feelings, changes in the oxyhemoglobin in the bilateral PFC during silent recall of negative episodes were significantly larger than those during the silent recall of positive episodes [65]. These findings may open an interesting avenue for future investigations about whether the non-involvement of the MPFC in the prioritisation of negative emotion can serve as an indicator of self-positivity.

Third, our functional connectivity analysis with the vmPFC as a seed did not reveal differences in connectivity between the self and positive emotion effects. A seed-to-voxel analysis with implemented advanced tools for removing potential confounding effects is susceptible to detecting small signal changes [66]. As such, we would detect the differences in synchronised activity between the MPFC and the rest of the brain if prioritisation of self and positive emotion recruit distinct neural networks. However, we did not observe the differences even with a lower threshold. Comparing self and negative biases demonstrated increased coupling between the MPFC and the left insula, right frontal opercular, right cuneus, and left middle occipital gyrus. This finding is in line with a vast body of works investigating neural mechanisms involved in the processing of self-relevant information. For example, it was suggested that co-activation between the insula involved in the salience network and areas involved in the DMN (such as the MPFC) is crucial in constituting the self and assigning self-specificity to stimuli [67]. This suggestion is supported by evidence from a meta-analysis showing the recruitment of the right IFG and the left insula during self-specific conditions [68] and in a study using both internally and externally cued conditions in a self-referential task [16]. Interestingly, a recent line of research raised the question of the specific engagement of DMN areas in self-referential processing, and more generally, how the flow of external information to DMN subregions is regulated. The most prominent finding in this work indicated that self-referential processing elicited information flow from the fronto-parietal areas to support task-specific information processing [69]. For example, ROI-based analysis in a recent fMRI study showed that the activity in the vmPFC negatively correlated with the activity in the dorsolateral prefrontal cortex (DLPFC) in self-referential judgments [40]. Our functional connectivity results of anticorrelation between the MPFC and fronto-parietal areas (right frontal opercular, and right cuneus) support this work.

Taken together, our data demonstrate distinct and non-overlapping neural properties supporting the prioritisation of self-relevant information and negative emotion. Nevertheless, we did not find differences between self-prioritisation and positive emotion-prioritisation. This raises the question of what brain mechanisms may support the self-positivity effect? One emerging theory of emotions posits how we process emotions is deeply rooted in predictions that our brain constructs for incoming emotional information [70,71]. Therefore, the meaning of incoming emotional information is partly predetermined by the content of representations that the brain has formed about a concept of an emotional category. A positive concept of self is inherently associated with happiness [72]. This creates the possibility that seeing a correct pairing associated with happy emotion triggers representations associated with happiness and, as a consequence, activates the most strong representation in the MPFC—about the self. This explanation echoed converging lines of evidence [14,15,25,73] that the MPFC is not likely to be related to emotional content per se but is due to the self-referential processing of emotional stimuli.

In contrast, seeing a shape associated with a sad expression may trigger representations of a concept formed about negative emotions (e.g., sadness). When representations of negative emotions are not linked to the self, they may recruit a different brain network such as the frontal and parietal areas found in the present study, which are known to relate to sadness [74] and negative emotions in general [75,76]. However, it is important to note that our experimental design included only happy and sad emotions and therefore, does not allow for a firm conclusion about the whole range of negative emotions.

We also cannot exclude an alternative explanation for the self-positivity bias. Seeing a positive emotional expression can be rewarding. The link between happy emotions and reward as motivational factors is well-documented in behavioural, electrophysiological, and neuroimaging studies [77]. There is also evidence for similarity in behavioural and neural effects of self and reward. For example, an enhanced response to reward and the self [78] has been repeatedly observed in conjunction with elevated neural activations in cortical midline structures such as the vmPFC [79]. Although the literature provides mixed findings of whether reward, self, and emotion processing overlap [78,80], we cannot exclude the possibility that the interplay between these three systems determines the self-positivity bias in the present study.

In conclusion, we report that the prioritisation effects for self and positive emotions was shown to be tightly linked together through the MPFC, and that the MPFC played a role in discriminating between positive and negative emotions in relation to self-relevance. We propose that the experimental framework of associative matching could provide a useful means of systematically mapping self-prioritisation and emotion prioritisation effects.

## Figures and Tables

**Table 1:** Significant clusters for contrasts in the emotion task.

Contrast	Label	x	y	z	k	Z
happy > neutral	Precentral_L	−26	−22	64	289	3.67
	Parietal_Inf_l	−40	−38	40	543	4.65
sad > neutral	Frontal_Inf_Tri_L	−54	14	28	421	4.49
	Supp Motor Area_L	−2	2	62	141	4.31
	Frontal Sup_2_L	−24	0	64	306	4.09
	Frontal_Inf_Tri_R	46	16	22	207	3.93

False discovery rate (FDR)-corrected at cluster level *p* < 0.05, height threshold *p* < 0.001, and uncorrected, extended threshold at 30 voxels.

**Table 2:** Significant clusters for differences in functional connectivity between the vmPFC and the rest of the brain for self-biases compared to sad-biases.

	x	y	z	k	t-Value
Left Insula *	−28	16	16	187	9.31
Right frontal inferior opercular *	46	−04	04	103	−5.72
Right cuneus *	24	−96	10	61	−5.51
Left occipital middle gyrus	−14	−90	14	48	−5.44

* The results remained significant even with more conservative threshold of FDR with *p* < 0.001.

## Supplementary Materials

The following are available online at https://www.mdpi.com/2076-3425/11/2/264/s1. Table S1: MASQ scores for participants in the present sample, Figure S1: Means percent correct responses in the personal (A) and emotion (B) tasks for matched (correct pairings) and mismatched (incorrect pairings) associations between shapes and labels, Table S2: The effects of Stimulus on accuracy performance, Figure S2: Means reaction time in the personal (A) and emotion (B) tasks for matched (correct pairings) and mismatched (incorrect pairings) associations between shapes and labels, Table S3: The effects of Stimulus on Response Times, Table S4: Comparison RT advantages, Figure S3: Residuals vs. Predicted and Q-Q Plot Standardized, Table S5: Cluster for contrast happy > neutral above the threshold, Figure S4: Activation results for the whole-brain univariate analyses for contrasts [happy > neutral], Table S6: Clusters for contrast sad > neutral above the threshold, Figure S5: Activation results for the whole-brain univariate analyses for contrasts [sad > neutral], Table S7: Clusters for contrast self > friend (height threshold *p* < 0.005, extended threshold = 30), Figure S6: The ROIs defined in the present study.
