# Supplementary material for: Self-Positivity or Self-Negativity as a Function of the Medial Prefrontal Cortex
Source: Brain Sci. 2021 Feb 19;11(2):264. doi: 10.3390/brainsci11020264 (PMC7922957; doi:10.3390/brainsci11020264)
Supplement: Supplementary file 1 [file brainsci-11-00264-s001.pdf]

Supplementary Materials

Pre-screening

One-day prior to the scanning session, participants were sent the Mood and Anxiety Symptom Questionnaire (Watson et al., 1995). The MASQ is a 77-item self-report questionnaire that assesses General Distress: depressive (12 items), anxious (11 items) and mixed symptomatology (15 items). The questionnaire also included an anxiety-specific (Anxious Arousal, 17 items) and a depression-specific scale (Anhedonic Depression, 22 items). Higher scores reflect greater levels of symptomatology. The reported internal consistency for each scale is excellent with coefficient alphas ranging from 0.78 to 0.92. Factorial validity for the MASQ has been established in non-clinical samples, with three factors consistently found to best represent the data (Geisser et al., 2006; Reidy et al., 1997).

We used cut-off points based on Watson et al (1995, Table 1)

Table 1
Descriptive Statistics for the MASQ Scales in Five Subject Groups

	Students		Adults		
MASQ scale	Male (n = 438)	Female (n = 635)	Male (n = 142)	Female (n = 186)	Patients (male; n = 453)
GD: Mixed					
M	34.5_a	35.2_a	31.3_b	$33.0_{a,b}$	34.9_a
SD	9.0	9.2	10.0	10.2	12.3
GD: Anxiety					
M	$22.3_{a,b}$	22.6_a	20.5_b	$20.8_{a,b}$	$21.6_{a,b}$
SD	6.4	6.3	7.5	6.7	7.5
Anxious Arousal					
M	27.8_a	27.1_a	24.4_b	24.2_b	28.3_a
SD	9.4	8.2	8.0	7.8	10.4
GD: Depression					
M	$24.5_{b,c}$	25.8_b	22.1_c	$25.0_{b,c}$	28.0_a
SD	8.7	8.8	8.1	9.4	10.0
Anhedonic Depression					
M	55.6_b	54.2_b	52.0_b	55.2_b	65.5_a
SD	13.4	13.9	12.5	15.2	14.8

Note. Within a row, means not sharing a subscript are significantly different from one another ($p < .05$, two-tailed). MASQ = Mood and Anxiety Symptom Questionnaire; GD = General Distress.

The MSAQ questionnaire was emailed to each participant. For each item, they indicated to what extent they had experienced each symptom (1 = not at al, 5 =

extremely) during the past week including today. Participants were asked to return their completed answer list in the same day. Scores for each participants on the present study are presented in Table 1S.

Table 1S. MASQ scores for participants in the present sample. Two participants (marked in red) were not admitted to the scanning session. GD = General Distress

ID	GD: Mixed	GD: Anxiety	Anxious Arousal	GD: Depression	Anhedonic Depression
1	32	22	26	24	46
2	30	18	23	22	44
3	32	24	24	23	49
4	28	16	19	20	47
5	36	25	28	27	52
6	30	20	23	21	48
7	39	27	28	22	54
8	42	28	25	27	55
9	35	22	24	25	54
10	32	21	22	21	53
11	32	22	22	25	49
13	34	23	27	27	50
14	32	22	21	22	48
15	33	25	22	20	47
16	32	24	20	22	51
17	37	27	25	45	58
18	35	29	23	25	53
19	38	28	28	37	51
21	32	23	21	25	48
22	33	24	19	26	54
23	29	19	23	20	48
12	46	32	29	48	58
20	52	42	29	48	59
**Mean (SD)***	**33.48 (3.4)**	**23.29 (3.4)**	**23.48 (2.8)**	**25.05 (5.9)**	**50.43 (3.5)**

*Mean and SD are based on participants data who were invited to a scanning session

Behavioural performance

Matched and mismatched pairs were analysed separately due to different responses being made in these cases.

Accuracy

The overall accuracy in responding to stimuli in the personal (Fig.S1, A) and emotion (Fig.S1, B) tasks was above 85%.

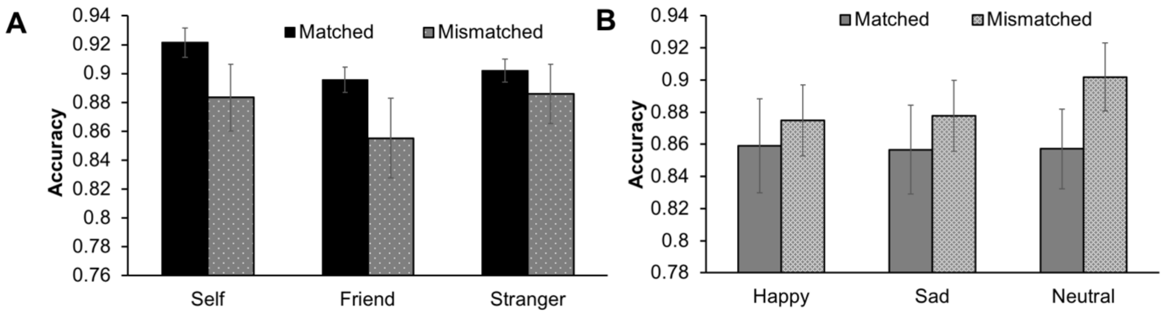

Fig. S1. Means percent correct responses in the personal (A) and emotion (B) tasks for matched (correct pairings) and mismatched (incorrect pairings) associations between shapes and labels. The error bars represent +/-SEM.

A one-way repeated measures ANOVA was carried out on accuracy data for matched and mismatched trials in each task. The results of these analyses are summarised in Table S1.

Table S2. *The effects of Stimulus on accuracy performance*

	**Personal task**	
Matched	A main effect<br>F(2,40)=3.62,<br>p=0.036,<br>$\eta^2 = 0.07$	*Post Hoc<br>***Self vs Friend***<br>$t(20)= 2.58$, p_h =.04, MD= 0.03, 95% CI [0.009; 0.041]<br>***Self vs Stranger***<br>$t(20)= 1.96$, p_h = .12, MD= 0.02, 95% CI [-0.003; 0.042]<br>***Friend vs Stranger***<br>$t(20)= -0.62$, p_h = .54, MD= -0.01, 95% CI [-0.028; 0.0165]
Mismatche d	F(2,40)=3.22,<br>p=0.05,	***Self vs Friend***<br>$t(20)= 2.10$, p_h = .08, MD= 0.03, 95% CI [-0.005; 0.061]

	◎^{2} = 0.02	***Self vs Stranger***<br>t(20)= -0.18, p_h = 0.86, MD= -0.002, 95% CI [-0.026; 0.021]<br>***Friend vs Stranger***<br>t(20)= -2.28, p_h = 0.84, MD= - -0.03, 95% CI[-0.055; -0.00]
	**Emotion task**	
Matched	F(2,40)=0.015,<br>p=0.98,<br>◎^{2} = 0.00	Post Hoc<br>***Happy vs Sad***<br>t(20)= 0.16, p_h =1.0, MD= 0.0<br>***Happy vs Neutral***<br>t(20)= 0.13, p_h =1.0, MD= 0.0<br>***Sad vs Neutral***<br>t(20)= -0.03, p_h =1.0, MD= 0.0
Mismatched	F(2,40)=0.66,<br>p=0.52,<br>◎^{2} = 0.02	Post Hoc<br>***Happy vs Sad***<br>t(20)= -0.11, p_h =.91, MD= -0.0<br>***Happy vs Neutral***<br>t(20)= -1.05, p_h =.91, MD= -0.03<br>***Sad vs Neutral***<br>t(20)= -0.94, p_h =.91, MD= -0.02

*Post Hoc: paired sample *t*-test. We report the adjusted p-value for multiple comparisons using Holm Method (Holm, 1979). This method, in a stepwise way, computes the significance levels depending on the p-value based rank of hypotheses. MD- difference in means (the alternative hypothesis for these tests was that a true difference in means is not equal to 0).

A paired sample t-test showed that the differences in accuracy between matched and mismatched trials in personal and emotion tasks were non-significant (t(20)=1.42, p=.69; MD=0.031; 95%CI[-0.05; 0.08] and (t(20)= -1.68, p= 0.11; MD= -0.03; 95%CI [-0.06; 0.01] for personal and emotion tasks respectively).

Reaction time

Means reaction time are displayed in Fig.S2

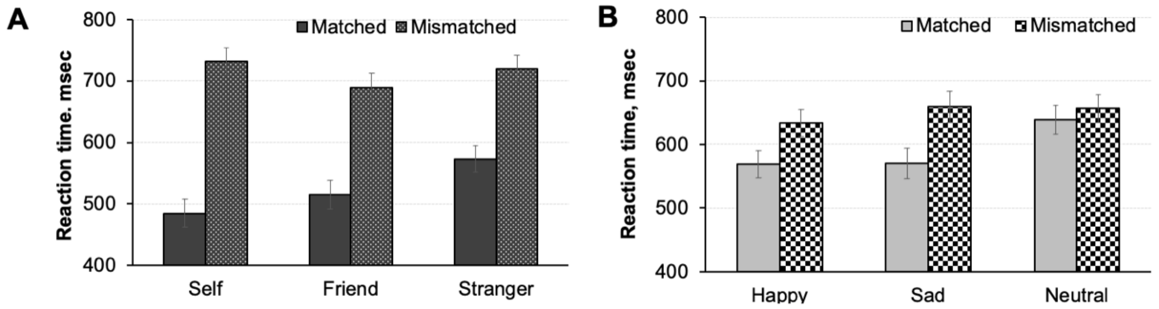

Fig. S2. Means reaction time in the personal (A) and emotion (B) tasks for matched (correct pairings) and mismatched (incorrect pairings) associations between shapes and labels. The error bars represent +/-SEM.

A one-way repeated measures ANOVA was carried out on RT data for matched and mismatched trials in each task. The results of these analyses are summarised in Table S2.

Table S3. *The effects of Stimulus on Response Times*

	**Personal task**	
Matched	A main effect<br>F(2,40)= 27.62,<br>p<0.001,<br>◎^{2} = 0.12	Post Hoc<br>***Self vs Friend***<br>t(20)= -2.54, p_h = .02, MD= -30.58, 95% CI [-60.37,- 2.84]<br>***Self vs Stranger***<br>t(20)= -7.32, p_h < .001, MD= -88.24, 95% CI [-100.1; -72.71]<br>***Friend vs Stranger***<br>t(20)= -4.78, p_h < .001, MD= -57.66, 95% CI [-82.98; -32.33]
Mismatched	F(2,40)=27.96,<br>p<0.001,<br>◎^{2} = 0.17	***Self vs Friend***<br>t(20)= 7.22, p_h < .001, MD= 42.63, 95% CI [31.32; 53.93]<br>***Self vs Stranger***<br>t(20)= 1.91, p_h = .06, MD= 11.28, 95% CI [-0.79; 23.35]<br>***Friend vs Stranger***<br>t(20)= -5.31, p_h < .001, MD= -31.35, 95% CI [-44.84; -17.85]
	**Emotion task**	

Matched	F(2,40)=29.70,<br>p<0.001,<br>◎^{2} = 0.10	***Happy vs Sad***<br>*t*(20)= -0.32, p_h = .75, MD= -3.24, 95% CI [-11.88; 10.36]<br>***Happy vs Neutral***<br>*t*(20)= -6.83, p_h < .001, MD= -69.38, 95% CI [-84.93; -47.35]<br>***Sad vs Neutral***<br>*t*(20)= -6.51, p_h < .001, MD= -66.14, 95% CI [-82.97; -47.78]
Mismatched	F(2,40)=13.64,<br>p<0.001,<br>◎^{2} = 0.05	***Happy vs Sad***<br>*t*(20)= -4.73, p_h < .001, MD= -25.22, 95% CI [-35.36; -15.06]<br>***Happy vs Neutral***<br>*t*(20)= -4.29, p_h < .001, MD= -22.85, 95% CI [-36.92; -8.77]<br>***Sad vs Neutral***<br>*t*(20)= 0.44, p_h = .07, MD= 2.37, 95% CI [-6.00; 10.73]

A paired sample t-test showed that participants were faster in responding to matched trials compared to mismatched in the personal task (*t*(20)= -9.16, p <.001; MD= -188.71; 95%CI [-231.67; -145.75]. The difference between matched and mismatched trials in the emotion task did not reach significance (*t*(20)= -2.01, p= 0.06; MD= -54.84; 95%CI [-111.81; 2.11].

Reaction Time Advantages

To quantify the effects of personal relevance and emotions, we calculated the advantage in RT for self and friend compared to stranger ([RTstranger – RTself], [RTstranger – RTfriend]) and happy and sad emotional expressions compared to neutral ([RTneutral – RThappy], [RTneutral – RTsad]) (Table S3).

Table S4. *Comparison RT advantages*

Contrast	Results (Student's t-test)
[RTstranger – RTself] - [RTstranger – RTfriend]	*t*(20)=2.70, p=.01, MD = 39.39, SE difference = 14.54, 95%CI [8.95,

	69.62], Cohen's d =0.59, 95%CI [0.12, 1.05]
[RTneutral – RThappy] - [RTneutral – RTsad]	*t*(20)=0.29, p=.78, MD = 3.24, SE difference = 11.34, 95%CI [-20.43, 26.90], Cohen's d =0.06, 95%CI [-0.37, 0.49]

Multiple regression

A multiple regression analysis was carried out to test whether the magnitude of the prioritization effects for positive and negative emotions could predict the magnitude of self-prioritization. Using the enter method it was found that prioritization of positive and negative emotions explained a significant amount of the variance of self-prioritization (F(2,20) = 9.04, p=0.002, R^2 = 0.50). The model summary and test statistics are presented below (all analyses were performed in JASP, 2020).

Model Summary

Model	R	R^2	Adjusted R^2	RMSE
H_0	0.000	0.000	0.000	31.437
H_1	0.708	0.501	0.446	23.403

ANOVA

Model		Sum of Squares	df	Mean Square	F	p
H_1	Regression	9906.980	2	4953.490	9.044	0.002
	Residual	9858.829	18	547.713		
	Total	19765.810	20			

Note. The intercept model is omitted, as no meaningful information can be shown.

Bootstrap Coefficients

Model		Unstandardized	Bias	Standard Error	95% bca* CI	
					Lower	Upper
H_0	(Intercept)	88.429	0.187	6.660	74.667	100.963
H_1	(Intercept)	46.141	-0.759	12.408	27.080	77.519
	Happy-bias	0.421	0.033	0.148	0.170	0.699
	Sad-bias	0.179	-0.015	0.141	-0.112	0.430

* Bias corrected accelerated

Note. Bootstrapping based on 5000 replicates.

Note. Coefficient estimate is based on the median of the bootstrap distribution.

In order to check for outliers, we assessed residual statistics (see table below). An analysis of standard residuals was carried out, which showed that the data contained no outliers (Std. Residual Min = -1.93, Std. Residual Max = 1.55).

Residuals Statistics

	Minimum	Maximum	Mean	SD	N
Predicted Value	50.589	138.296	88.238	22.256	21
Residual	-41.888	32.983	-7.615e -16	22.202	21
Std. Predicted Value	-1.692	2.249	3.192e -16	1.000	21
Std. Residual	-1.930	1.553	-0.012	1.039	21

Tests to see if the data met the assumption of collinearity indicated that multicollinearity was not a concern (Happy-bias, Tolerance = .92,VIF = 1.09; Sad-bias, Tolerance = .92,VIF= 1.09) (see table below).

Coefficients

Model		Unstandardized	Standard Error	Standardized	t	p	95% CI		Collinearity Statistics	
							Lower	Upper	Tolerance	VIF
H_0	(Intercept)	88.238	6.860		12.862	< .001	73.928	102.548		
H_1	(Intercept)	47.502	11.371		4.178	< .001	23.613	71.391		
	Happy-bias	0.410	0.119	0.598	3.434	0.003	0.159	0.661	0.915	1.092
	Sad-bias	0.186	0.132	0.244	1.402	0.178	−0.093	0.464	0.915	1.092

The data met the assumption of independent errors (Durbin-Watson value = 2.63) (see table below).

Model Summary

Model	R	R^2	Adjusted R^2	RMSE	Durbin-Watson Autocorrelation	Durbin-Watson Statistic	Durbin-Watson p
H_0	0.000	0.000	0.000	31.437	-0.435	2.719	0.083
H_1	0.708	0.501	0.446	23.403	-0.413	2.628	0.161

The balanced distribution of the residuals around the baseline (Fig. S3) suggests that the assumption of homoscedasticity has not been violated. The Q-Q plot (Fig. S3) shows that the standardized residuals fit along the diagonal suggesting that both assumptions or normality and linearity have also not been violated.

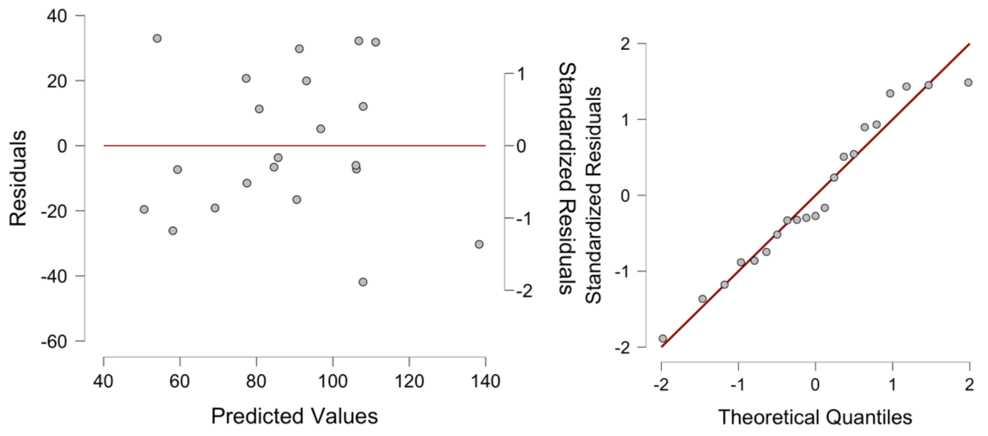

Fig. S3. Residuals vs. Predicted (on the right) and Q-Q Plot Standardized Residuals

Univariate fMRI

The contrast [happy > neutral] (height threshold p<0.001, extended threshold = 30 contagious voxels, cluster FDR corrected <0.05) showed activation in the left precentral lobule (Table S4, Fig.S4). No voxels survived the threshold for the reverse contrast [neutral > happy].

Table S5. Clusters for contrast happy > neutral above the threshold.

Label	x	y	z	k	Z
L-Precentral	-26	-22	64	289	3.67

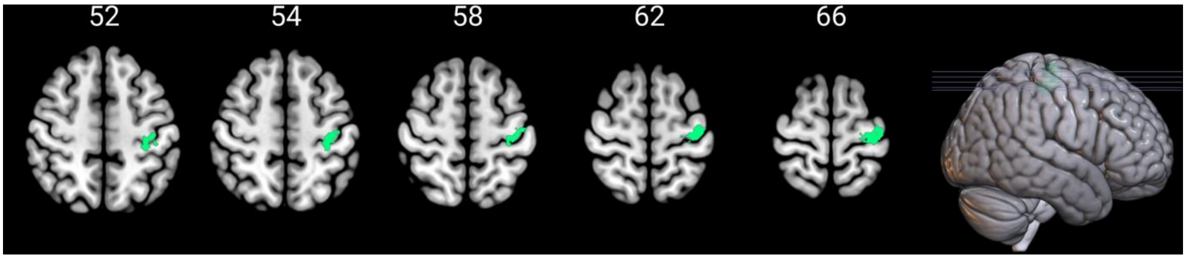

Fig.S4. Activation results for the whole-brain univariate analyses for contrasts [happy>neutral]. The mask of clusters with significant univariate effects (a cluster corrected FDR-threshold of p<0.05, voxel-threshold p <0.001 uncorrected, extended threshold of 30 contiguous voxels) was created and overlaid on a MNI152 standard template using MRIcroGL (radiological convention)

The contrast [sad > neutral] (height threshold p<0.001, extended threshold = 30 contagious voxels, cluster FDR corrected <0.05) showed activation in the left precentral lobule (Table S5, Fig.S5). No voxels survived the threshold for the reverse contrast [neutral > sad].

Table S6. Clusters for contrast sad > neutral above the threshold.

Label	x	y	z	k	Z
Parietal_Inf_l	-40	-38	40	543	4.65

Frontal_Inf_Tri_L	-54	14	28	421	4.49
Supp Motor Area_L	-2	2	62	141	4.31
Frontal Sup_2_L	-24	0	64	306	4.09
Frontal_Inf_Tri_R	46	16	22	207	3.93

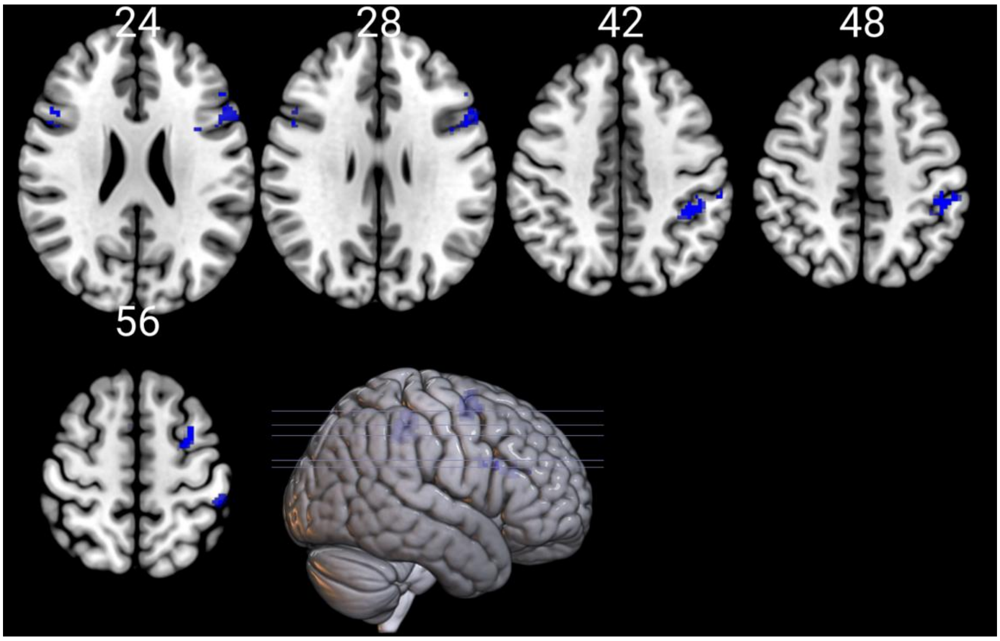

Fig.S5. Activation results for the whole-brain univariate analyses for contrasts [sad>neutral]. The mask of clusters with significant univariate effects (a cluster corrected FDR-threshold of p<0.05, voxel-threshold p <0.001 uncorrected, extended threshold of 30 contiguous voxels) was created and overlaid on a MNI152 standard template using MRIcroGL (radiological convention).

In the contrast [self > friend] (height threshold p<0.001, extended threshold = 30 contagious voxels, cluster FDR corrected <0.05) no voxels survived the threshold. Lowering the hight threshold to p<0.005 indicated activation in the MPFC (Rectus) and Precuneus. (Table S6). However, the activations were not significant at the FDR corrections.

Table S7. Clusters for contrast self > friend (height threshold p<0.005, extended threshold =30)

Label	x	y	z	k	Z	FDR_corr
Rectus	-2	-52	18	116	3.53	0.86
Precuneus	4	52	-18	82	3.22	0.86

The reverse contrast [friend > self] did not reveal voxels above the threshold (height threshold p<0.001, extended threshold = 30 contagious voxels, cluster FDR corrected <0.05). Lowering the height threshold to p<0.005 indicate clusters in the left dorsolateral prefrontal cortex (DLPFC) (x/y/z = -28/36/10, k=104, Z =4.03) and small clusters in the left parietal cortex. However, the activations were not significant at the FDR corrections.

Defining a ROI in the dmPFC

The dmPFC ROI was defined as a 7 mm sphere centred at x/y/z = 6/44/18 [41, 53] and containing 207 voxels (Fig S6).

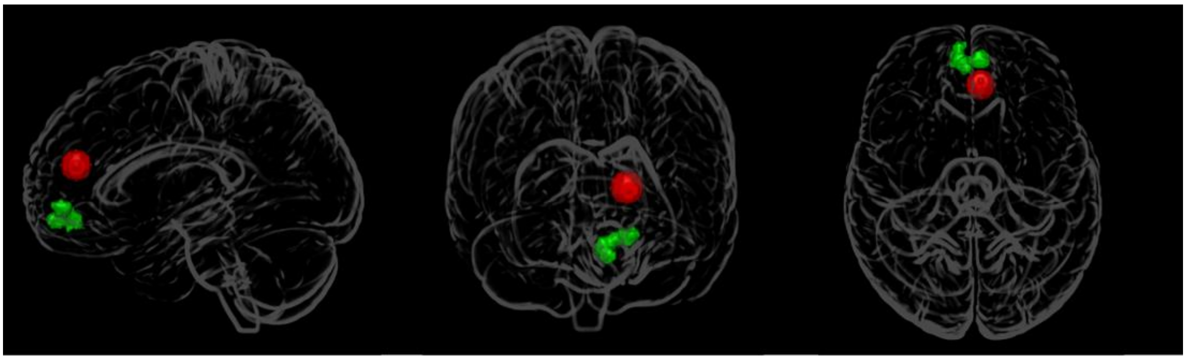

Figure S6. The ROIs defined in the present study. The vmPFC (in green) is the ROI defined based on the contrast [self > stranger] (k= 213, centred at x/y/z = -6/52/-4).

The dmPFC (in red) is the ROI defined based on previous studies (Mitchel at al., 2006) and Yankouskaya et al., 2018) (7 mm sphere centred at x/y/z = 6/44/18, k=207).

References for Supplementary Materials

1. Watson D, Clark LA, Weber K, Assenheimer JS, Strauss ME, McCormick RA.(1995). Testing a tripartite model: II. Exploring the symptom structure of anxiety and depression in student, adult, and patient samples. Journal of Abnormal Psychology, 104, 15–25. doi: 10.1037/0021-843X.104.1.15
2. Geisser ME, Cano A, Foran H. Psychometric properties of the Mood and Anxiety Symptom Questionnaire in patients with chronic pain. Clinical Journal of Pain. 2006;22:1–9. doi: 10.1097/01.ajp.0000146180.55778.4d.
3. Reidy J, Keogh E. (1997). Testing the discriminant and convergent validity of the Mood and Anxiety Symptoms Questionnaire using a British sample. Personality and Individual Differences, 23, 337–344. doi: 10.1016/S0191-8869(97)00048-2.
4. Holm M. (1979). A simple sequentially rejective multiple test procedure. *Scand J Statist, 6*, 65-70.
5. Mitchell, J.P., Macrae, C.N., & Banaji, M.R. (2006). Dissociable medial prefrontal contributions to judgments of similar and dissimilar others. Neuron, 50(4), 655-663. doi: 10.1016/j.neuron.2006.03.040
6. Yankouskaya, A., Humphreys, G., Stolte, M., Stokes, M., Moradi, Z., & Sui, J. (2017). An anterior-posterior axis within the ventromedial prefrontal cortex separates self and reward. *Social cognitive and affective neuroscience*, *12*(12), 1859–1868. https://doi.org/10.1093/scan/nsx112
